# Feasibility of Self-Performed Lung Ultrasound with Remote Teleguidance for Monitoring at Home COVID-19 Patients

**DOI:** 10.3390/biomedicines10102569

**Published:** 2022-10-13

**Authors:** Emanuele Pivetta, Anna Ravetti, Giulia Paglietta, Irene Cara, Federico Buggè, Gitana Scozzari, Milena M. Maule, Fulvio Morello, Stefania Locatelli, Enrico Lupia

**Affiliations:** 1 Division of Emergency Medicine and High Dependency Unit, Città della Salute e della Scienza di Torino, Molinette Hospital, 10126 Turin, Italy; 2 Department of Medical Sciences, University of Turin, 10126 Turin, Italy; 3 Residency Program in Emergency Medicine, University of Turin, 10126 Turin, Italy; 4 Città di Torino Local Health Unit and Out-of-Hospital Care Special Unit, 10126 Turin, Italy; 5 Hospital Medical Direction, Città della Salute e della Scienza di Torino, Molinette Hospital, 10126 Turin, Italy; 6 Cancer Epidemiology Unit and CPO-Piemonte, Città della Salute e della Scienza di Torino, Molinette Hospital, 10126 Turin, Italy

**Keywords:** telemedicine, COVID-19, lung, ultrasonography

## Abstract

During the COVID-19 pandemic, use of telemedicine with the aim of reducing the rate of viral transmission increased. This proof-of-concept observational study was planned to test the feasibility of a home-based lung ultrasound (LUS) follow-up performed by patients with mild COVID-19 infection on themselves. We enrolled patients presenting to the emergency department with SARS-CoV-2 infection without signs of pneumonia and indication to discharge. Each patient received a brief training on how to perform LUS and a handheld ultrasound probe. Then, patients were contacted on a daily basis, and LUS images were acquired by the patients themselves under “teleguidance” by the investigator. Twenty-one patients were enrolled with a median age of 44 years. All evaluations were of sufficient quality for a follow up. Probability of a better LUS quality was related to higher degree (odds ratio, OR, 1.42, 95% CI 0.5–3.99) and a lower quality to evaluation time (from 0.71, 95% CI 0.55–0.92 for less than 7 min, to 0.52, 95% CI 0.38–0.7, between 7 and 10 min, and to 0.29, 95% CI 0.2–0.43, for evaluations longer than 10 min). No effect related to gender or age was detected. LUS performed by patients and remotely overseen by expert providers seems to be a feasible and reliable telemedicine tool.

## 1. Introduction

Telemedicine, literally “healing at a distance”, is a way of delivering health care services by exchanging medical information from one site to another using Information and Communication Technologies. Compared to other types of remote patients’ monitoring, telemedicine requires both audio and visual components. Thanks to interactive telemedicine, clinicians and patients are able to exchange information in real-time, mainly by using digital cameras or self-monitoring devices. Telemedicine is not conceived to replace the traditional health services or to limit the physician–patient relationship but to improve the efficiency of healthcare by playing an important role in prevention, diagnosis, treatment, and follow-up [1,2].

It has different aims, from allowing access to specialist evaluation that could not otherwise be possible to offering the patient a frequent and closer monitoring [3]. Some experiments were conducted on the International Space Station [4,5,6].

During the COVID-19 pandemic, the number of telemedicine evaluations increased following the period-specific restrictions, in order to reduce the rate of viral transmission and, at the same time, in order to allow chronic patient to maintain the usual follow-up evaluation rate. In the lockdown period, telemedicine played this important role and demonstrated to be a possible tool to carry on, in some cases, the new “outpatient clinic evaluations”, mostly when elective medical visits had to be delayed [3,7].

Ultrasound is a diagnostic tool used since the fifties, at the beginning only in the obstetric field, due to the absence of radiation exposure risk, and then in the cardiological one [8]. In a few years, the ultrasound has become very popular in different settings. It is generally performed by ad hoc trained specialists, mainly radiologists, but during the last twenty years, it has also been performed by other physicians (e.g., those working in emergency medicine and critical care) [9,10].

Training is always the main issue in ultrasound use. Along with technical limits, the quality of the obtained images remains the biggest limitation of such a diagnostic tool.

However, some studies demonstrated the opportunity of obtaining appropriate images by non-specialist providers and, in appropriate contexts, even by non-physicians (e.g., nurses, physical therapists, or physician assistants) [11,12,13].

In the present study, we tested the feasibility of a home-based lung ultrasound (LUS) follow-up performed by the patients themselves in cases of mild COVID-19 infection without a clinical indication of hospital admission.

## 2. Materials and Methods

In this proof-of-concept observational study, we planned to enroll COVID-19 patients presenting to the emergency department (ED) of the Città della Salute e della Scienza di Torino University Hospital. All of them reported symptoms suspected for SARS-CoV-2 infection and were tested positive for SARS-CoV-2 at RT-PCR performed on nasopharyngeal swab samples but had neither signs of interstitial pneumonia at chest X-ray, LUS, or CT scan; nor oxygen need; nor indication of hospital admission. Moreover, they needed to have a Wi-Fi connection at home.

**Figure 1:**
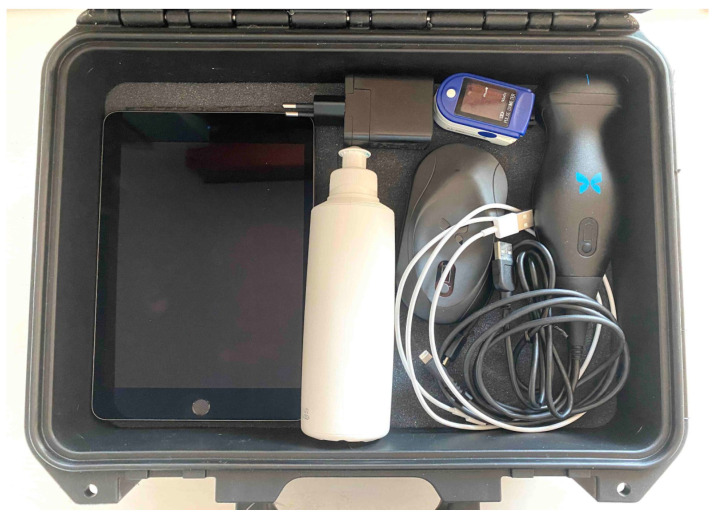
Home sonographic evaluation kit (see the Methods section for details).

All enrolled patients received a box with a handheld ultrasound probe (Butterfly iQ, Butterfly Network Inc, Guildford, CT, USA), sonographic gel, a tablet (iPad 4, Apple Inc, Cupertino, CA, USA), and a 1-finger oximeter (Figure 1).

Starting from the first day after ED discharge, patients were contacted on a daily basis for a maximum of 8 days or until the need of an in presence clinical evaluation, in hospital. All of them had the possibility of bringing forward the end of the follow-up. At the time of enrolment, each patient received a brief explanation of how to perform the lung ultrasound (LUS) evaluation along with a printed schematic summary.

The LUS ultrasound evaluations were performed using a 12-zone protocol [14,15,16].

Briefly, for each hemi-thorax, we evaluated 2 anterior, 2 lateral, and 2 posterior zones, exploring the upper and lower lung parenchyma (divided by the inter-nipple line).

The remote monitoring was performed using a feature of the handheld ultrasound probe, called “teleguidance”. At previously arranged times, the patients contacted the investigator online for the scheduled daily examination. By using the function “teleguidance”, he had the possibility to not only explain to the patient where to position the probe, but to also adjust preset, gain, depth, and remotely record videos and still images.

All evaluations were performed using a lung preset (3 MHz), collecting videos for a minimum of 6 s per area.

The entity of parenchymal disease was assessed using the LUS score [17,18].

Along with the sonographic images, every day we collected symptoms, oxygen saturation, evaluation time, and need for additional help in performing LUS evaluation.

The study design was approved by the Città della Salute e della Scienza di Torino University Hospital institutional review board (No. 00115173), and it was conducted in accordance with the principles of the Declaration of Helsinki for clinical research involving human subjects. All patients or their substitute decision makers provided informed consent.

Descriptive results are presented as median and interquartile range (IQR) for continuous data or as number and percentage for ordinal data.

We assessed intra- and inter-rater agreement in evaluating patients’ self-performed LUS scanning quality between two offline reviewers and two staff emergency physicians with expertise in LUS by using Cohen’s kappa. The two offline reviewers were two residents in emergency medicine, already trained in LUS and who had performed at least 200 live examinations at the time of the study (I.C. and G.P.), whereas the two staff emergency physicians with expertise in LUS (A.R. and E.P.) had more than 1000 LUS performed. The level of agreement was evaluated using the logical interpretation suggested by McHugh in 2012 [19].

We used a multilevel model for dichotomous outcome in order to assess the probability of obtaining a sonographic image of adequate quality (2–3 vs. 0–1) based on some independent variables either related to patient characteristics (i.e., age, gender, education) or to LUS examination itself (i.e., evaluation length and day, pulmonary field examined, and need of help) [20].

Due to the proof-of-concept study design, we did not perform a sample size calculation, and we planned to enroll 20 patients to test the research hypothesis.

Data were collected with an Excel spreadsheet (version 16.43; Microsoft, Redmond, WA, USA), and analyses were conducted with Stata (version 17.0/SE; StataCorp, College Station, TX, USA).

## 3. Results

Between December 2019 and May 2020, we enrolled 21 patients (10 women), with a median age of 44 years (range 12–61 years). Four patients (19.1%) were active smokers, 5 suffered from mild-moderate obesity (23.8%), 2 reported ongoing treatment for hypertension (9.5%), and 4 for dyslipidemia (19.1%).

About half of enrolled patients got a first level degree (52.44%).

All materials were used for the study only and not removed from the hospital. Moreover, no material was either spoiled or lost. All probes, chargers, and iPads were cleaned at the time of return to the research team.

During the study period, none of them needed to be admitted for worsening of the COVID-19 infection; all of them were then tested negative and totally recovered from the infection.

**Figure 2:**
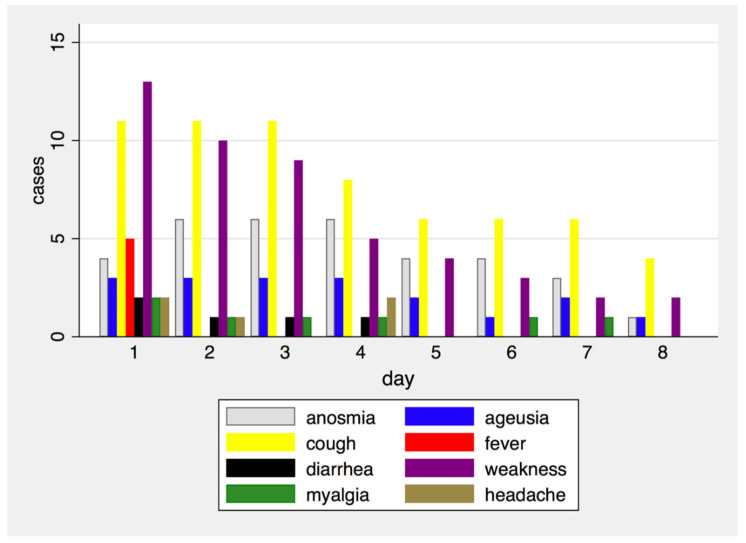
Frequency of symptoms by study day.

During the first day of evaluation, the most reported symptoms were cough (52.4%) and weakness (61.9%). Figure 2 shows symptoms’ frequency during each day of the study; in particular, shortness of breath was never reported.

**Figure 3:**
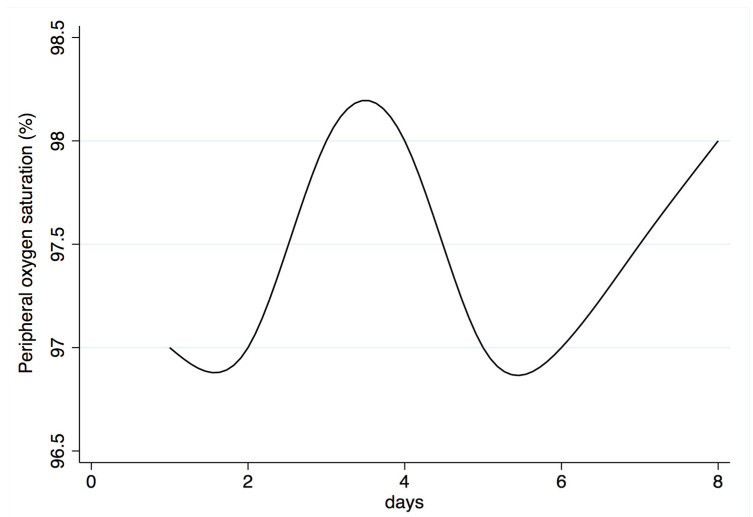
Peripheral oxygen saturation distribution by study day.

At the first day of evaluation, median peripheral oxygen saturation was 97% (range 93–99%). Figure 3 shows its evolution during the following days until the end of the study period.

**Figure 4:**
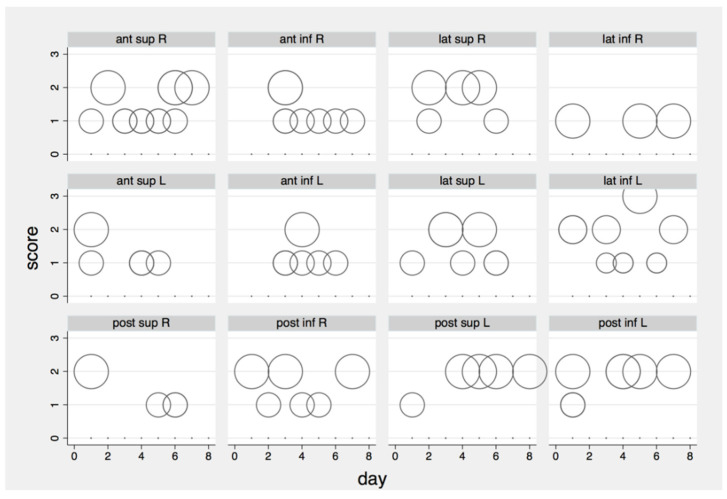
Abnormal LUS score per area and follow up day (ant, anterior; lat, lateral; post, posterior; sup, superior; inf, inferior; L, left; R, right; a larger dot symbolizes more patients).

**Figure 5:**
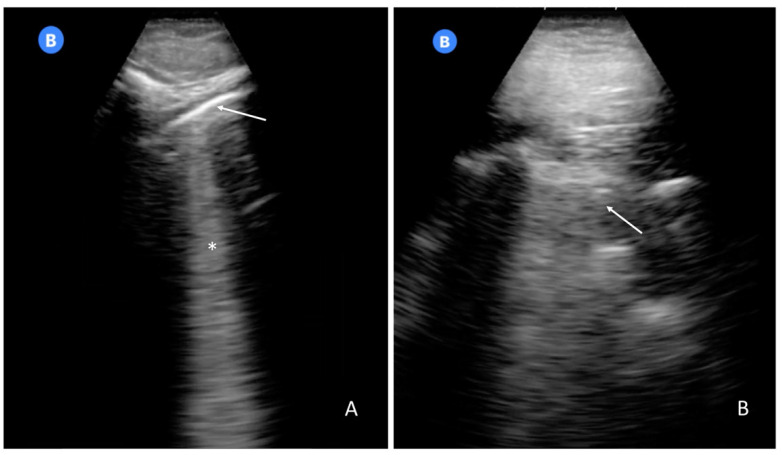
Most frequent findings in LUS self-performed by patients. Panel (**A**) shows a normal pleural line (arrow) and a B-line (asterisk—16). Panel (**B**) shows an irregular pleural line (see the arrow—16).

Figure 4 shows the no zero (i.e., abnormal lung parenchyma) LUS scores reported per scanning area during the entire follow-up period, as evaluated offline by one of the expert emergency physicians. In total, a few areas per day were abnormal: 14 in day 1, 4 in day 2, 14 in day 3, 16 in day 4, 12 in day 5, 13 in day 6, 6 in day 7, and 1 in day 8. The most frequent US artifacts found were B-lines and pleural line irregularities and the overall median LUS score was 0 (range 0–2—Figure 4 and Figure 5).

The median time for LUS evaluation was 13 min (range 8–17), and 10 patients (47.6%) needed the help of a relative or a cohabitant at least once during the study period.

Table 1 shows the median of all LUS acquisition quality assessments, as they were offline evaluated by the two reviewers.

Considering the independent variables related to LUS evaluation/performance itself, the model showed a significant effect of the time needed to perform LUS on the probability to obtain high quality LUS images (OR0.71, 95% CI 0.55–0.92 for LUS evaluations shorter than 7 min, OR 0.52, 95% CI 0.38–0.7, for LUS evaluation between 7 and 10 min, and OR 0.29, 95% CI 0.2–0.43, for LUS evaluations longer than 10 min)

On the contrary, we observed neither a significant effect of the scanning areas examined (OR 0.96, 95% CI 0.78–1.18 for the lateral areas, OR 1.06, 95% CI 0.89–1.26, for the posterior areas) nor of the presence of help during LUS performance (OR 0.98, 95% CI 0.58–1.61).

**Figure 6:**
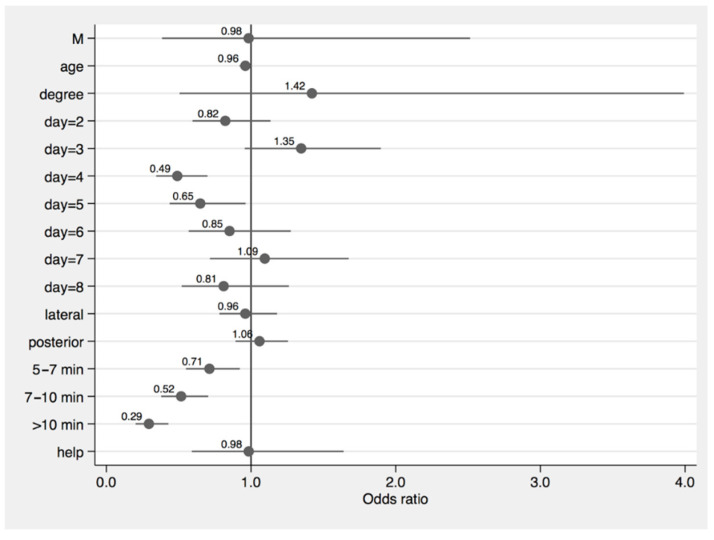
Effects of several independent variables on the probability of obtaining good quality images during LUS evaluation (M: male vs. female).

Finally, when we considered the day of follow-up in which LUS was performed, this showed an OR of 0.82 (95% CI 0.6–1.13) in day 2, 1.34 (95% CI 0.96–1.9) in day 3, 0.49 (95% CI 0.34–0.7) in day 4, 0.65 (95% CI 0.44–0.96) in day 5, 0.85 (0.57–1.27) in day 6, 1.1 (95% CI 0.72–1.67) in day 7, and 0.81 (95% CI 0.52–1.26) in day 8 (Figure 6).

Inter-rater agreement, estimated on the first offline revision of 1740 scanned areas between emergency medicine staff and resident reviewers in evaluating LUS quality was between 80 and 100% and between 69.70 and 100%, respectively.

Intra-reviewer agreement, evaluated between emergency medicine residents, was between 62.96 and 100% for each LUS area.

## 4. Discussion

During the COVID-19 pandemic, Italian hospitals and especially EDs had to deal with dramatic overcrowding, due not only to the rapid viral spread but also to some additional difficulties of our healthcare system [21,22,23], including the lack of a good prevention system and of well-functioning territorial healthcare services and the insufficient number of healthcare workers [24]. In this situation, telemedicine can represent an important tool to help facing these difficulties.

The present study obtained positive results on the feasibility of a home-based LUS follow-up, performed by COVID-19 patients themselves and interpreted by remotely connected physicians, and analyzed possible factors influencing its performance.

The quality of images is the most relevant limitation on the use of ultrasounds even when this is performed by healthcare workers, and this concern is even greater when US evaluation is performed by less trained operators.

In our study, most of the LUS videos collected by the patients (only 5.9% of 728 zones evaluated were categorized as of low quality) had an adequate quality to enable physicians to properly evaluate the lung parenchyma. This evaluation was possible not only for the physician who was remotely connected and assisted the patient in “real-time” during LUS performance but also for a second physician, who reviewed the same images later, “offline”. Moreover, the LUS score obtained by different reviewers showed a good concordance degree, even when re-evaluated by physicians with lower expertise in LUS.

Therefore, limited instructions provided to patients seem sufficient to obtain a correct probe placement and the recording of good quality LUS images. However, it must be underlined that, in our study, LUS interpretation was always made by the physician who remotely assisted the patient during LUS performance and not by patients themselves.

In addition, the sonographic evaluation was not the only component of the remote follow-up evaluated in the present study. All patients were, indeed, asked to report symptoms, body temperature, and oxygen saturation daily. The results obtained suggest that the combination of remote monitoring of clinical symptoms and LUS images allows the researchers to perform an effective follow-up and probably to reduce the need for clinical re-evaluation, in particular new ED evaluations. None of the enrolled patients required, indeed, new clinical “in person” evaluations, and all of them totally recovered from COVID-19, although this might be also at least partially related to the mild form of the disease that affected all enrolled patients.

We can speculate that, during the pandemic surges, the implementation of a similar approach to patient care could allow the healthcare systems a more effective organization. In particular, surges like that due to the omicron variant, which appears more contagious but less virulent [25], put a large number of people in home quarantine [26,27,28]. In a similar situation, out-of-hospital healthcare providers will be likely insufficient to guarantee the requested number of home evaluations for each patient needed to promptly identify a worsening situation. The telemedical approach could be one potential answer from a national healthcare system to this need.

Our study confirms and extends the results reported by Kirkpatrick and colleagues in a similar study performed on the International Space Station examining self-performed telemonitored LUS. These authors involved 27 self-isolated healthy subjects with previous exposition to ultrasound but without direct experience in performing LUS and showed that the quality of collected LUS images was high and allowed a valid online lung evaluation [29].

Compared to this study [29], a strength of our study surely resides in having enrolled “real” patients without previous knowledge of ultrasound use and in a difficult “real world” situation, namely, in the ED during a pandemic.

However, some issues emerged during the study that need to be discussed.

The main problem we met during the carrying out of the present study was due to technical difficulties, in particular related to the stability of internet connection or to transient malfunctioning of devices.

None of the evaluations had to be canceled due to this issue, which accounted for longer evaluation times. However, this problem could be of greater importance, for instance, in rural areas (our study was totally held in Turin, a metropolitan area in the northwest of Italy). Similarly, the availability of a good quality internet connection had an impact on patients’ selection, and this could therefore affect the generalizability of our results. More studies need to be conducted in particular to assess whether our results would be confirmed in larger cohorts and among patients less used to technology.

Moreover, it is reasonable to assume that a similar out-of-hospital approach to the follow-up of COVID-19 patients is possible only in regions where a network of emergency medical services is present, and patients can be quickly evaluated and hospitalized if necessary.

In addition, COVID-19 presentation and deterioration patterns are also quite characteristic and easy to identify with LUS, helping physicians in deciding about possible hospitalization need. Since other pathologies (e.g., heart failure) share with interstitial pneumonia a similar LUS pattern, it can be tempting to assume that patients affected by these pathologies can also managed with a similar follow-up approach. However, ad hoc studies are needed to evaluate whether this approach is feasible and safe in these cases. As this is a proof-of-concept study, our sample size was only 21 patients, similar to the population enrolled in Kirkpatrick’s study [29]. Our patients were quite young (median age of 44 years) and had a high level of education and few comorbidities. This could represent an additional limitation for the generalizability of the results of our study, since patients admitted to the ED are often older and with a lower level of education. However, this model of remote follow-up could be at least employed for the follow-up of younger patients or of those more accustomed to using technological supports, representing a potential useful tool for reducing ED overcrowding.

It is also interesting that the present follow-up approach was feasible also in the case of a twelve-year-old child, with parental help. Although additional studies are needed, it can be hypothesized that this approach would be reliable, assuming that the parents of pediatric patients have features, in terms of age and technology habit, similar to those of the patients enrolled in the present study [30]. A potential limitation of our study was that the offline evaluators of LUS images were members of the research team. Although, in our study, all revisions were blindly performed, reviewers might have been more exposed to overestimate the quality of images. This possible limitation needs to be tested in further studies.

The multilevel model used for evaluating those factors influencing the probability of obtaining good quality LUS images underlined the expected impact of education and evaluation time. In our study, a longer time needed for LUS assessment was probably related to more difficult LUS evaluation and, as a consequence, to poorer quality images. The results referring to the day in which LUS was performed could be explained by a higher attention when performing LUS in the first days, probably related to the higher concern about the possible COVID-19 evolution, whereas, in the following days, when the clinical situation was stable and/or improving, patient’s attention dropped. Finally, the null effect of the presence of help is likely due to the features of our patients and relatives: since none of them had previous medical training, help in performing LUS was equally difficult with and without it.

## 5. Conclusions

In conclusion, the use of LUS performed by patients themselves remotely overseen by expert providers seems to be a feasible and reliable telemedicine tool useful in treating COVID-19 patients. Images achieved using this protocol might be of good quality and usable for remote follow-up. The quality of LUS images seems to be related to the time needed for LUS performance and to the number of evaluations performed but not to the patient’s degree, gender, age, or the eventual presence of any help in performing LUS.

The results of our study have to be confirmed in larger cohorts and, potentially, extended to patients with diseases other than COVID-19.

## Figures and Tables

**Table 1:** Overall evaluation of LUS quality by the 2 reviewers per each scanning area.

	POSTERIOR LEFT	LATERAL LEFT	ANTERIOR LEFT	ANTERIOR RIGHT	LATERAL RIGHT	POSTERIOR RIGHT
SUPERIOR	3 (1–3) 3 (1–3) 3 (1–3) 3 (1–3) 3 (1–3) 3 (1–3) 3 (1–3) 3 (1–3)	2 (1–3) 3 (1–3) 3 (1–3) 3 (1–3) 2 (1–3) 2 (1–3) 2 (1–3) 2 (1–3)	3 (1–3) 3 (1–3) 3 (0–3) 2 (1–3) 3 (1–3) 3 (1–3) 3 (1–3) 3 (1–3)	2 (1–3) 3 (1–3) 3 (1–3) 2 (1–3) 2 (1–3) 2 (1–3) 2 (1–3) 3 (1–3)	3 (1–3) 2 (1–3) 2.5 (1–3) 2 (1–3) 2 (1–3) 3 (1–3) 3 (1–3) 2 (1–3)	2 (1–3) 3 (1–3) 3 (2–3) 3 (1–3) 3 (1–3) 3 (1–3) 3 (1–3) 3 (1–3)
INFERIOR	3 (1–3) 3 (1–3) 3 (2–3) 2 (1–3) 3 (1–3) 3 (1–3) 3 (1–3) 3 (1–3)	2 (1–3) 3 (1–3) 2.5 (1–3) 2 (1–3) 3 (1–3) 2 (1–3) 2 (1–3) 2 (1–3)	2 (1–3) 3 (1–3) 2 (1–3) 3 (1–3) 2 (1–3) 3 (1–3) 2.5 (1–3) 3 (1–3)	2 (1–3) 2 (1–3) 2 (1–3) 2 (1–3) 2 (1–3) 2 (1–3) 2 (1–3) 3 (2–3)	2 (1–3) 3 (1–3) 2 (1–3) 2 (1–3) 2 (1–3) 2 (1–3) 3 (1–3) 2 (1–3)	3 (0–3) 3 (1–3) 3 (1–3) 3 (1–3) 2 (1–3) 3 (1–3) 3 (1–3) 3 (2–3)

The multilevel model for dichotomous outcome (expressed as a quality of LUS images of 2–3 vs. 0–1) showed an OR of 1.42 (95% CI 0.5–3.99) for patients with a degree, of 0.98 (95% CI 0.38–2.51) for male patients, and of OR 0.96 (95% CI 0.92–1.00) for age unit increase.

## Data Availability

Not applicable.
